# 3D Porous Polycaprolactone with Chitosan-Graft-PCL Modified Surface for In Situ Tissue Engineering

**DOI:** 10.3390/polym17030383

**Published:** 2025-01-30

**Authors:** Johannes Pitts, Robert Hänsch, Yvonne Roger, Andrea Hoffmann, Henning Menzel

**Affiliations:** 1 Institute for Technical Chemistry, Braunschweig University of Technology, 38106 Braunschweig, Germany; 2 Institute of Plant Biology, Braunschweig University of Technology, 38106 Braunschweig, Germany; 3 Hannover Medical School, Department of Orthopaedic Surgery, Biological Basics for Biohybrid Implants, 30625 Hannover, Germany; 4 Niedersächsisches Zentrum für Biomedizintechnik, Implantatforschung und Entwicklung (NIFE), 30625 Hannover, Germany

**Keywords:** in situ tissue engineering, PCL, polymer blending, PEO, sucrose, surface modification, surface potential, porosity, macroporous scaffold, chitosan-graft-PCL, TGF release, in vitro cell testing

## Abstract

Tissue engineering has emerged as a promising approach for improved regeneration of native tissue and could increase the quality of life of many patients. However, the treatment of injured tissue transitions is still in its early stages, relying primarily on a purely physical approach in medical surgery. A biodegradable implant with a modified surface that is capable of biological active protein delivery via a nanoparticulate release system could advance the field of musculoskeletal disorder treatments enormously. In this study, interconnected 3D macroporous scaffolds based on Polycaprolactone (PCL) were fabricated in a successive process of blending, annealing and leaching. Blending with varying parts of Polyethylene oxide (PEO), NaCl and (powdered) sucrose and altering processing conditions yielded scaffolds with a huge variety of morphologies. The resulting unmodified hydrophobic scaffolds were modified using two graft polymers (CS-g-PCL_x_) with x = 29 and 56 (x = PCL units per chitosan unit). Due to the chitosan backbone hydrophilicity was increased and a platform for a versatile nanoparticulate release system was introduced. The graft polymers were synthesized via ring opening polymerization (ROP) of ε-Caprolactone using hydroxy groups of the chitosan backbone as initiators (grafting from). The suspected impact on biocompatibility of the modification was investigated by in vitro cell testing. In addition, the CS-g-PCL modification opened up the possibility of Layer by Layer (LbL) coating with alginate (ALG) and TGF-β_3_-loaded chitosan tripolyphosphate (CS-TGF-β_3_-TPP) nanoparticles. The subsequent release study showed promising amounts of growth factor released regarding successful in vitro cell differentiation and therefore could have a possible therapeutic impact.

## 1. Introduction

Subsequent to mental disorders, musculoskeletal disorders are the second highest contributor to disability, with a global prevalence of 1.3 billion in 2017 and rising, leading to 136 million years lived with disability (YLDs) in total after a 20% increase during the last decade [1]. Therefore, reducing treatment duration as well as YLDs, while improving the outcome of necessary surgical repairs, is key to improving patients’ quality of life and addressing the significant socio-economic impact of musculoskeletal disorders. The complexity of multi-tissue units in the musculoskeletal system and its interfaces make treatment a challenge.

Today, state-of-the-art surgical treatments of various parts of the human body rely predominantly on physical approaches. Examples of this are techniques like suture anchor repairs, autograft transfers and the cutting of bones in osteotomy in general [2,3,4,5].

These techniques have in common that no active healing support is provided at the trauma site post-surgery. While autografts at least provide patient-specific scaffolds, they tend to be limited by the donor site morbidity and scarcity of autologous and allogeneic tissue. Here, the field of tissue engineering (TE) could provide a solution as it does not suffer from most of these major limitations [6,7,8,9]. TE offers the custom tailoring of a scaffold according to the needs of the targeted tissue as well as the application of specific active agents at the trauma site. In addition, tissue-engineered scaffolds or biomaterials have to fulfill numerous requirements specific to the targeted tissue and trauma type. Requirements to consider are of biological, morphological, and mechanical nature, as well as the degradability of the chosen material [10,11,12]. Ideally, the material supports the specific tissue by providing physical and biochemical assistance while being vascularized until the natural healing processes of the human body have remedied the trauma or are able to continue on their own. The simultaneous degradation of the scaffold ensures that no re-surgery of the patient is needed. Depending on the type of application, this degradation time has to be adjusted [13,14,15,16,17]. For proper vascularization, a minimum pore diameter of 100–250 µm is needed [18,19].

Custom-tailored scaffolds based on synthetic polymers satisfy many of the demanded requirements while being cost-efficient and available in abundance. While natural polymers in most cases are regarded as superior when it comes to biological and mechanical properties, they have severe drawbacks like batch inconsistency and scarcity, often making them too unreliable for medical applications [20,21].

Polycaprolactone (PCL) is a well-known synthetic material in tissue engineering applications with melt- and electrospun fibers, 3D-printed scaffolds, interconnected 3D porous materials, and many more shapes [17,22,23,24,25,26,27,28,29]. Its high mechanical load capabilities and slow degradation speed in the human body make PCL a promising candidate for in situ tissue engineering [30]. However, without chemical modification, the hydrophobic nature of PCL makes initial cell attachment difficult and slows down cell proliferation, which hinders vascularization in vivo [31]. A possible way to increase hydrophilicity and biocompatibility of the PCL can be via plasma treatment or several other morphological, chemical, or biological modifications [32,33,34,35]. Modification on a chemical level can be performed with Chitosan (CS). CS is derived from Chitin which, after cellulose, is the second most abundant natural polymer in the world [36,37]. Due to its hydrophilicity, antibacterial properties, good biocompatibility, and degradability, Chitosan is used across a wide range of fields in biomedical, food packaging, water treatment, and pharmaceutical applications [38,39,40,41,42,43,44]. In tissue engineering chitosan is known to provide a variety of benefits. Examples are the promoted attachment and proliferation of osteoblasts and chondrocytes, as well as its capabilities as a drug delivery system [45,46,47]. The combination of CS and PCL by grafting PCL onto a CS backbone is already well known, and the resulting CS-g-PCL_x_ species can be tailored according to the ratio of PCL units per chitosan unit (x), which determines the properties of the copolymer [33,34,35]. Other PCL modification approaches like the covalent bonding to its surface or via blending often require the modification step to be integrated in scaffold fabrication or, due to harsh chemical conditions, limit scaffold delicacy. 

Co-crystallization of the modification does not suffer from these limitations as it can be performed after scaffold fabrication under comparably mild conditions. However, modification by co-crystallization of the graft copolymer on the surface of PCL scaffolds is up to now only known in the field of delicate electrospun fiber mats and melt-spun textile fibers [29,33,34,48]. These scaffolds do not offer pores or interfiber spaces that fulfill the required 100–250 µm pore diameter for cell ingrowth and vascularization [18,19].

Scaffolds based on interconnected 3D porous PCL with CS-g-PCL modification could offer a substantial improvement over the lack of mechanical capabilities of their delicate electrospun competitors while still offering good biocompatibility and good vascularization [25,26]. Furthermore, the modification with CS-g-PCL offers the possibility of applying a layer-by-layer (LbL) coating with alginate (ALG) and TGF-β_3_-loaded chitosan tripolyphosphate (CS-TGF-β_3_-TPP) nanoparticles based on the electrostatic interactions of CS, ALG and TPP [33,34,35].

This nanoparticulate system, in combination with a scaffold that provides appropriately sized pores for vascularization, could promote cell ingrowth specific to the growth factor chosen. In addition to TGF-β_3_, which regulates the proliferation, differentiation and adhesion of cells, CS-TPP nanoparticles can encapsulate a variety of other pharmaceutically active substances as a drug release system. Examples are other growth factors like the bone-morphogenetic protein BMP-2, plasmid DNA or low molecular weight substances like curcumin [49,50,51]. This shows the high potential of a tailored porous PCL scaffold combined with such a nanoparticulate release system.

This study investigates the fabrication of interconnected 3D porous PCL obtained through a sequence of polymer blending, annealing, and leaching. Various compositions have been fabricated and evaluated regarding their feasibility as a possible implant material for in situ TE. For the first time, the surface of interconnected 3D porous PCL was modified with two chitosan graft polymers (CS-g-PCL_x_). The copolymers differ in their ratios x (x = PCL units per chitosan unit). Successful modification was proven via SEM, CLSM, and zeta potential measurements, and a suspected increase in cytocompatibility was investigated in in vitro cell testing. The modification enabled the introduction of a nanoparticulate chitosan TGF-β_3_ tripolyphosphate (CS-TGF-β_3_-TPP) release system. Subsequent in vitro release studies showed promising amounts of TGF-β_3_ released, suggesting a possible therapeutic impact.

## 2. Materials and Methods

### 2.1. Materials

#### 2.1.1. Fabrication of Interconnected 3D-Porous Poly(ε-caprolactone)

Poly(ε-caprolactone) (PCL, Mn = 80 kDa, Sigma Aldrich, Taufkirchen, Germany) was blended with polyethylene oxide (PEO, POLYOX™ WSR-N10, Mn = 100 kDa, DOW^®^ Chemical, Midland, MI, USA) in a Haake Rheomix OS by THERMO SCIENTIFIC™ (Waltham, MA, USA) equipped with a Banbury rotor. In addition to PEO, fine and powered sucrose (≥99,7%, Nordzucker, Braunschweig, Germany), and NaCl (≥99,5%, Fisher Scientific, Schwerte, Germany) were added to the blend in order to manipulate its morphology and, in some cases, to replace the PEO completely. The blending procedure was carried out according to Mehr et al. [42]. Before each batch, the mixer was flooded with nitrogen gas to prevent thermo-oxidative degradation. Temperature was set to 100 °C at 50 rpm rotation speed. For non-PEO-containing batches, the temperature was raised to 175 °C. The fill level during the 7 min passes was kept at 80 vol% (48/60 mL). After blending, the hot blend was immersed in liquid nitrogen to freeze the morphology. Table 1 shows the fabricated blend compositions.

The blends were annealed under nitrogen atmosphere at 160 °C for 3–6 h. Therefore, 20 g of a blend was put between two (15 × 15) cm^2^, 1 cm thick, aluminum plates with adjustable spacers (2 mm) in each corner. After annealing, the whole aperture was doused with liquid nitrogen until the plates were well below 0 °C.

The plates were removed and the obtained blends, discs with 10 cm in diameter, were immersed in deionized water (300 mL per disc) and gently stirred (200 rpm). Water was replaced thrice a day for the following 5 days. Finally, the porous scaffolds were dried in a vacuum oven for 24 h at RT. The final scaffolds were immersed in liquid nitrogen and cut-outs (16 × 8 × 2) mm^3^ were prepared with a razor knife. If not stated otherwise, these cut-outs were used in all consecutive modification and analysis steps.

#### 2.1.2. CS-g-PCL_x_ Synthesis

CS-g-PCL_x_ was synthesized via grafting polycaprolactone (PCL) from a chitosan (CS) backbone by the ring-opening polymerization of ε-caprolactone in a slightly modified procedure derived from de Cassan et al. [33] and Jing et al. [48].

Dry, purified Chitosan with a degree of deacetylation (DDA) of 83% (dried according to Gan et al. [52], 350 mg, 168.3 g/mol, 2.09 mmol, Mn = 190–310 kDa, Sigma Aldrich) was dissolved in methanesulfonic acid (2 mL, ≥99%, Sigma Aldrich) in a 250 mL dried Schlenk flask under a nitrogen atmosphere. CS was dissolved after 45 min at 50 °C. ε-Caprolactone CS was dissolved after 45 min at 50 °C. ε-Caprolactone monomer (5.3 mL, 56.16 mmol, 24 eq., ≥98%, Sigma Aldrich) was added to the solution and the mixture stirred for 5 h at 50 °C. Subsequently, an aqueous quench solution containing 0.2 M KH_2_PO_4_ (43.75 mL, ≥ 99.5 % Merck, Darmstadt, Germany), 10 M NaOH (7 mL, ≥97.0% Merck, Darmstadt, Germany), and 100 g of ice from deionized water was added. The precipitated crude CS-g-PCL was collected via centrifugation and vacuum dried for 48 h at RT.

The dried CS-g-PCL was dissolved in N,N-dimethylformamide (≥99%, Sigma Aldrich) and reprecipitated in ice. It was then (again) collected by centrifugation (10 min, 3500 rpm, 5 °C) and vacuum dried at RT for 48 h. Two different chitosan graft PCL copolymers (CS-g-PCL_x_) with CS to ε-caprolactone/PCL ratios (x) (PCL units per amino glucopyranose units in chitosan) were prepared. An overview of synthesis conditions for the two species used in this study can be found in Table 2. Catalyst/solvent and quenching chemicals were scaled linearly to the amount of CS used. Theoretical and calculated ratios x in CS-g-PCL_x_ were derived from stoichiometry and ^1^H-NMR integrals, respectively, via the method of Jing et al. [48] as shown in Section 3.2.

#### 2.1.3. Surface Modification of Interconnected 3D-Porous Poly(ε-caprolactone)

CS-g-PCL_x_ (0.5 wt%) was dissolved in acetic acid (aq, 81% (w/v)) at 50 °C for 1 h. The solution (15 mL per 5 scaffolds) was transferred into a petri dish (Ø = 50 mm) and allowed to cool to RT. The scaffolds were submerged in the solution for 2 min. Potential air trapped inside the scaffolds was removed by gentle shaking during the first 10 s of the procedure.

Afterwards, the scaffolds were removed from the solution, put into petri dishes, and placed into a vacuum oven at RT for 48 h. Finally, the scaffolds were washed with deionized water (300 mL per 10 scaffold cutouts) in three intervals of 8 h each, gently stirring as before during the leaching process. Subsequent drying for 48 h under a fume hood finalized the process. For modification with alginate, the CS-g-PCL_x_-scaffolds were dipped in an alginate solution (Milli-Q^®^, 5 mg/mL) for 2 min, and removed and rinsed with Milli-Q^®^ (Merck, Darmstadt, Germany). Therefore, the scaffolds were immersed in Milli-Q^®^ (300 mL each 10 scaffold cutouts and beaker) and gently stirred (50 rpm) for 30 min at RT. After removal from the beakers, the scaffolds were put into petri dishes and dried for 48 h under a fume hood at RT. Alginate fluoresceinamine modification for CLSM was performed similarly but with the alginate fluoresceinamine solution (Milli-Q^®^, 5 mg/mL) being filtered through a membrane filter (0.20 μm, polyamide, WHATMAN™, Freiburg, Germany). In addition, washing with Milli-Q^®^ was performed until the supernatant no longer emitted fluorescent light when looked at in near darkness under a handheld UV lamp (366 nm).

With Milli-Q^®^ being exchanged thrice per day, this took 5 d on a laboratory shaker (250 rpm, RT) in 45 mL centrifuge tubes filled to 35 mL with 5 scaffolds per tube. Non-modified samples also underwent this procedure in order to evaluate the necessity of CS-g-PCL modification for the alginate modification.

### 2.2. Methods

#### 2.2.1. Scanning Electron Microscopy (SEM)

All measurements were carried out on an EVO LS 25 by ZEISS (Oberkochen, Germany) with a voltage of 15 kV applied. Before the measurements, all samples were gold-sputtered.

#### 2.2.2. Mercury Intrusion Porosimetry

All porosimetry measurements were executed using a mercury intrusion porosimeter PoreMaster 60 by QUANTACHROME INSTRUMENTS (Boynton Beach, FL, USA). All measurements were performed in triplicate, collecting data on the intrusion and extrusion parameters of the (1.5 × 0.5 × 0.2) cm^3^ samples.

#### 2.2.3. X-Ray Microscopy (XRM)

All measurements were conducted on a Xradia 520 Versa by Zeiss (Oberkochen, Germany) with a flat-panel detector. Pixel size was 13.675 µm, with an exposure time of 0.2 s. Each image was averaged from 10 separate exposures. Voltage was set to 40 kV with an average power of 3 W. Binning was set to 1 with 3201 projections total.

Data evaluation was conducted with Dragonfly^©^ (build 2022.2.0.1399) by ORS (Montreal, Quebec, Canada) using lower and upper Otsu algorithms for void and material segmentation, respectively, and OpenPNM plugin analysis.

#### 2.2.4. Gel Permeation Chromatography (GPC)

GPC measurements were performed on a PSS SECcurity^2^ instrument equipped with a SECcurity^2^ vacuum degasser, a SECcurity^2^ TCC6500 column oven, an Agilent Infinity 1200 isocratic pump, a PSS SECcurity^2^ 1200 refractive index detector (relative measurement against polystyrene standard), and a manual injection valve from Rheodyne. All species were recorded in THF at 40 °C on a PSS triple SDV column setup (1× pre-column, 2× main columns, each 10 µm particle and pore size) with a concentration and flow rate of 1 g/L and 1 mL/min, respectively. All equipment was bought from Agilent (Santa Clara, CA, USA).

#### 2.2.5. Differential Scanning Calorimetry (DSC)

DSC measurements were performed on a DSC 204 thermal analysis device with a CC 200 cooling controller and a TASC 414/3 A system controller by NETZSCH (Selb, Germany). Samples were analyzed in aluminum crucibles (25/40 μL) with punctured lids (Thepro, Heinsberg, Germany). For gravimetrical measurements, a microbalance type XS3DU by METTLER TOLEDO (Giessen, Germany) was used. All measurements were performed in triplicate with a 10 K/min heating and cooling rate with 3 cycles per sample.

#### 2.2.6. Proton Nuclear Magnetic Resonance (^1^H-NMR)

All ^1^H-NMR spectra were obtained using an AV III 600 Spectrometer by BRUKER (Billerica, MA, USA). A magnetic field strength of 14.1 T at 600 MHz was applied to the in CDCl_3_ dissolved samples at RT. TMS was used as an internal standard in the series 528 5 mm high-field tubes by Sigma Aldrich^®^ (Taufkirchen, Germany). Data evaluation was carried out using MestReNova-Software Version 6.0.2-5475 by MESTRELAB (Santiago de Compostela, Spain).

#### 2.2.7. Confocal Laser Scanning Microscopy (CLSM)

CLSM was carried out on a ZEISS CLSM-510 Meta scan head connected to an Axiovert 200 M (Oberkochen, Germany). Measurement included multiple scans in various depths of the CS-g-PCL_56_/alginate fluoresceinamine scaffolds with 5× magnification at 488 nm excitation and 528 nm emission wavelength. Gain was set to 766 with a pinhole setting of 3.94 AU/76 µm. In addition, lambda scans were performed to verify that the detected light was a result of fluorescence and not a reflection of any other source. Data were processed with ZEN lite 3.7 (blue edition) by ZEISS (Oberkochen, Germany).

#### 2.2.8. Surface Potential

Surface potential measurements were performed on a SurPASS 3 by Anton Paar (Dahlewitz, Germany) in a pH range of 9.5 to 2. For 3D porous PCL, a cylindrical cell for porous materials was used. Each measurement was conducted with two (2 mm thick) discs pressed against each other. For solid PCL (2 × 1 × 0.2) cm^3^ and electrospun fiber mats (2 × 1) cm^2^ a gap cell was used instead, with two samples on each side of the cell.

#### 2.2.9. Loading and Release of TGF-β_3_

Modification with TGF-β_3_-loaded CS-TPP nanoparticles was performed in a dip coating procedure according to a previously published method [29,33,53,54]. For this purpose, TGF-β_3_ (10 µg/mL in 10 mM citric acid, stored at −80 °C) was dissolved in a CS solution (CS with a DDA of 42%, freeze-dried according to Gan et al. [52], in a solution of 1 mg/mL, acetic acid 0.1% (v/v) in Milli Q^®^) and the resulting solution was mixed with TPP (Milli-Q^®^, 1 mg/mL) in a 3:1 volume ratio regarding CS/TPP, yielding a CS-TGF-β_3_-TPP nanoparticle suspension containing 1 µg/mL TGF-β_3_.

Sterilized (see 2.5) CS-g-PCL_56_/Alginate-scaffolds were immersed under a clean bench in CS-TGF-β_3_-TPP nanoparticle suspension using Sorenson tubes (2 mL, low protein binding, Sorensen BioScience, Salt Lake City, Utah) for 20 min with one scaffold per tube. After removal from the tubes, the scaffolds were laid on low-lint paper towels to remove excess nanoparticle suspension. The scaffolds were washed twice by immersing them into tubes with MilliQ^®^ water. The scaffolds were removed from the tubes and excess water was removed using low-lint paper and successive vacuum drying at RT. All experiments were conducted with 4 samples per species, each within a separate tube.

The electrospun fiber mats ((8 × 16) mm^2^ cutouts), which serve as a reference benchmark, were incubated in groups of 4 per tube.

The TGF-β_3_-loaded scaffolds were immersed into Sorensen tubes (2 mL, low protein absorption, Sorensen BioScience, Salt Lake City, Utah) containing 1 mL PBS with BSA (0.1% (w/v)) and NaN_3_ (0.02% (w/v)). The release was performed at 37 °C in a heating cabinet with the supernatant being exchanged after 1, 8, 25, 96 h onwards until day 32 in 5-day intervals. Four samples per species were incubated and their supernatant was collected and stored at −20 °C for later ELISA analysis. In the case of the electrospun reference fiber mats, 4 samples were incubated in one tube.

#### 2.2.10. Enzyme-Linked Immunosorbent Assay (ELISA)

For quantification of the released human TGF-β_3_ in the supernatant, an ELISA kit ((DY243, 15 plates) and a duoset ELISA Ancillary Kit 2 (R&D System, Minneapolis, MN, USA) were used. The calibration was derived from a series of bulk protein solutions. As a readout, the absorbance of 3,3′,5,5′-tetramethylbenzinidine at 450 nm was detected using an Infinite^®^ 200 PRO (Tecan Group Ltd., Männedorf, Switzerland).

#### 2.2.11. Sterilization

Electron beam sterilization with an intensity of 25 kGy was conducted as described by de Cassan et al. [55]. The sterilization of the scaffolds was conducted by Mediscan (Kremsmünster, Österreich) according to EN ISO 13485 and ISO 11137. 

#### 2.2.12. Cultivation of Human Mesenchymal Stromal Cells

Human bone marrow-derived mesenchymal stromal cells (huBMSCs) were cultivated in growth medium (DMEM FG0415 from Biochrom; 10 % FCS Gibco from Thermo Fisher Scientific, Schwerte, Germany; 25 mM N-(2-hydroxyethyl)piperazine-N’-(2-ethanesulfonic acid (HEPES) from Carl Roth, Karlsruhe, Germany; 100 U/mL penicillin and 100 µg/mL streptomycin from Sigma Aldrich (Taufkirchen, Germany); 2 ng/mL recombinant human FGF-2 from Peprotech Now Thermo Fisher Scientific, Schwerte, Germany ) in tissue culture flasks at 37 °C and 5% CO_2_ until they reached a confluency around 70–80%. Cells were detached with trypsin/EDTA (Biochrom, MA, USA) and used for the vitality assay at a density of 10,000 cells per well.

Before seeding, the scaffolds were washed twice with PBS and once with growth medium at 37 °C and 5% CO_2_. After seeding, the scaffolds were incubated for 1, 3 and 7 days at 37 °C and 5% CO_2_.

#### 2.2.13. Actin Cytoskeleton and DNA Staining

After 3 days of incubation of huBMSCs on the scaffolds, the cells were stained with phalloidin-tetramethylrhodamine B isothiocyanate (phalloidin; cytoskeleton staining) and 4′,6-diamidino-2-phenylindole (DAPI; DNA staining) in order to analyze the morphology and distribution of the huBMSCs on the scaffolds. The cells were washed with PBS and fixated with 4% paraformaldehyde in PBS for 30 min at room temperature.

After another two washing steps with PBS, the cells were treated with 0.1% Triton X-100 in PBS for 10 min, followed by two washing steps with PBS. The cells were stained with phalloidin–tetramethylrhodamine B isothiocyanate (0.3 µM) and DAPI (1 µg/mL) for at least 1 h at room temperature in the dark. Finally, the cells were washed twice with PBS and kept in PBS in the dark until further analysis by microscopy (Olympus CKX53 incl. fluorescence kit CKX3-RFA, Olympus Europa, Hamburg, Germany)

#### 2.2.14. Vitality Assay

The vitality assay was performed according to the manufacturer’s manual (Promocell, Heidelberg, Germany) after 1, 3, and 7 days of incubation. In brief, used medium was removed from the well and replaced with fresh growth medium. A total of 1/10 of the WST8 reagent was added to each well and incubated at 37 °C and 5% CO_2_.

After four hours, 100 µL of the supernatant was transferred into a 96-well plate and the adsorption was measured at 450 nm with a plate reader. The results were processed via Excel (Version 2408, Microsoft, Washington, DC, USA).

## 3. Results

### 3.1. Interconnected 3D Porous Poly(ε-caprolactone)

Poly(ε-caprolactone) (PCL) was blended with polyethylene oxide (PEO)in an internal mixer according to Mehr et al. [42]. After blending, the hot blend was immersed in liquid nitrogen to freeze the morphology. Subsequently, the blends were annealed under controlled conditions and the PEO was leached by immersing the samples into water.

Processability of most blends displayed in Table 1 was satisfying, except blends with high contents of powdered sucrose (No. 12) and low-to-medium amounts of NaCl (No. 15–17). These were still processable but yielded slightly bridle granulose blends. With a high content of NaCl (No. 18) proper blending was not possible as the rotor could not get hold of the material anymore as the NaCl slipped from its metal surface, which can also be deduced from the torque diagrams. Blends with Chitosan-graft-PCL (CS-g-PCL_56_) were processable (No. **4**–**6**), however, the resulting materials were not applicable for later scaffold creation as they did not withstand leaching with water.

All further original blend compositions of the scaffold can be identified via the following pattern: X_Y_Z with X, Y, and Z as the vol.-% of PCL, PEO, and an additive, respectively. The additives “F” and “P” stand for fine sucrose and powdered sucrose, respectively.

**Figure 1:**
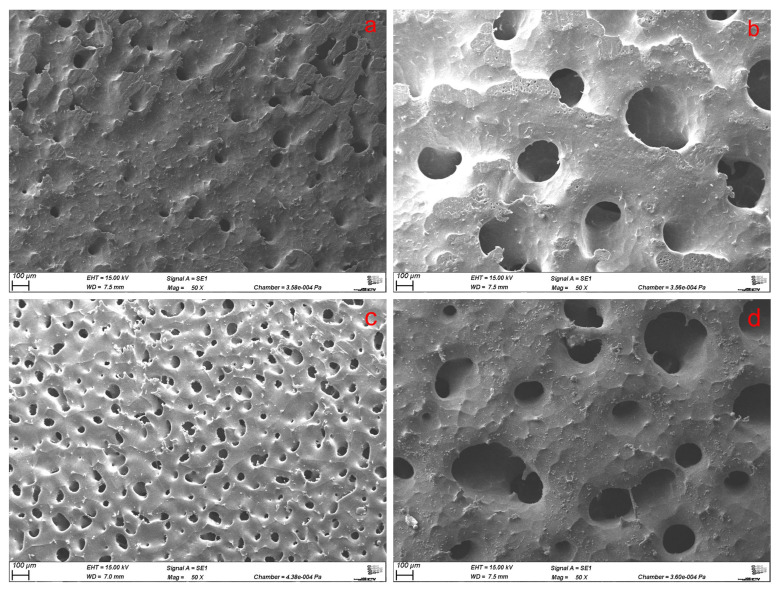
SEM images of 55_45 leached blends after (**a**) 3 h, (**b**) 6 h annealing and 45_55 leached blends after (**c**) 3 h, (**d**) 6 h annealing in 50x magnification (top view).

The optimal annealing duration to generate a co-continuous PCL/PEO network and therefore a decent interconnected porous scaffold after leaching was determined by annealing 55_45 and 45_55 blends up to 6 h. SEM of the leached blends revealed the desired interconnected network with an average pore size of approximately 250 µm, which was obtained after 6 h with both species showing similar pore sizes at first glance (Figure 1).

**Figure 2:**
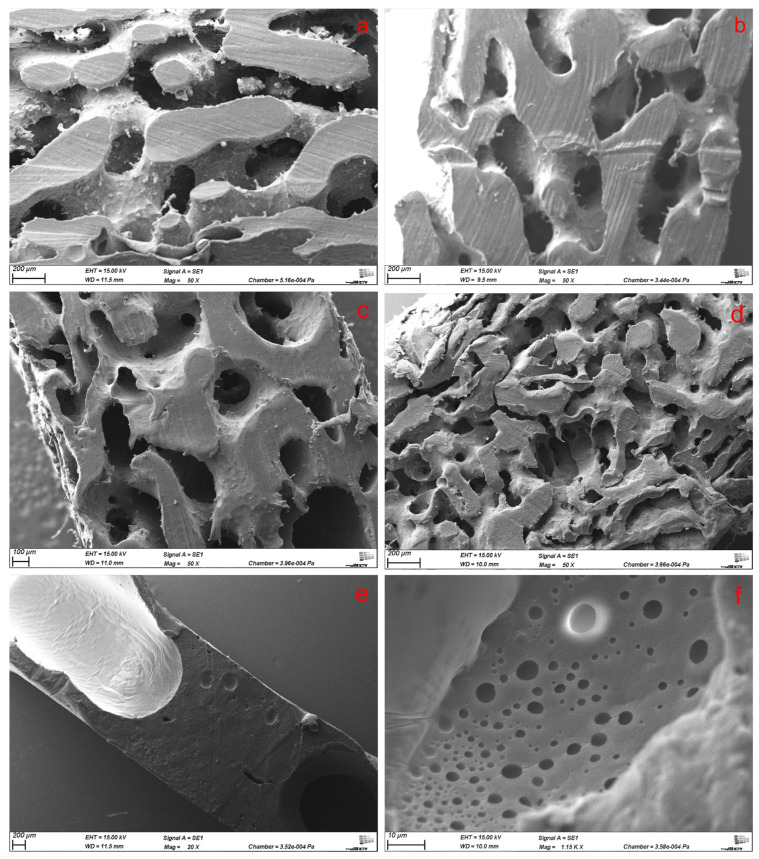
SEM images of cross sections from 2-mm-thick PCL scaffolds prepared from blends with (**a**) 45_55, (**b**) 50_50, (**c**) 45_45_10F, and (**d**) 45_45_10P composition at 50×magnification and 55_0_45F with (**e**) 20× and (**f**) 1150× magnification.

A slight orientation of the surface was visible, as well as some irregularities. These could originate from stripping of the aluminum plates after annealing, as it cannot be excluded that the PCL was still warm enough to undergo thermoplastic deformation. However, the SEM of cross sections of five different scaffolds revealed that the irregularities can even be found inside the network (Figure 2). The finest network structure, with eventually the largest surface area of all scaffolds, was found within material with powdered sucrose added (Figure 2d). The fine sucrose-added scaffold (Figure 2c) turned out to be shaped like the standard PCL/PEO-only scaffolds.

The complete replacement of PEO with fine sucrose (No. **9**) led to no formation of an interconnected network but showed fine micropores with diameters < 15 µm (Figure 2e,f). Species with sucrose and NaCl content above 10-vol.% had very low structural integrity and, therefore, were not further investigated.

**Figure 3:**
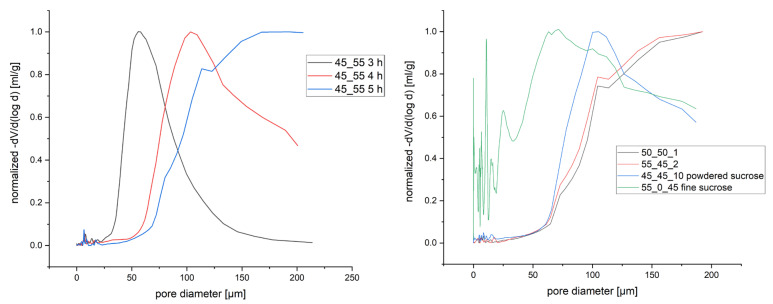
Normalized pore size distributions obtained via Hg porosimetry for different annealing times (**left**) and for different compositions after 6 h annealing time (**right**).

To quantify the observation made in SEM, the pore size distribution of several scaffolds was analyzed via Hg porosimetry (Figure 3). After 3 h annealing, the distribution of pores of a 45_55 scaffold was still in the measuring range of the porosimeter, peaking at 56 µm (Figure 3 left). This peak shifts to 103 µm after 4 h, with the larger pore sizes of the distribution not being measurable as the minimal pressure of the porosimeter only allows measurement of pores smaller than 215 µm. This trend continues; after 5 h annealing time almost solely pores with more than 100 µm were formed. Moving on to the results (right) for the 50_50 and 55_45 scaffolds with 6 h annealing time, this shift is even more pronounced with possible peak values cut of the distribution for the reason mentioned above. The 45_45_10P scaffold, on the other hand, shows smaller pore sizes compared to the non-sucrose-containing species with similar annealing times. Their peak and distribution are more like a 4-h-annealed 45_55 variant. The 55_0_45F scaffold (green), prepared without PEG but only with finely powdered sucrose, shows a distribution shifted to even lower values. This could come from the fact that most of the bigger pores had expanded beyond the 2-mm thickness of the samples and, therefore, cannot be properly measured. However, the interesting point here is the quantitative evaluation of the smaller pores already observed in SEM (Figure 2f). These smaller pores were not found for any other scaffold.

**Figure 4:**
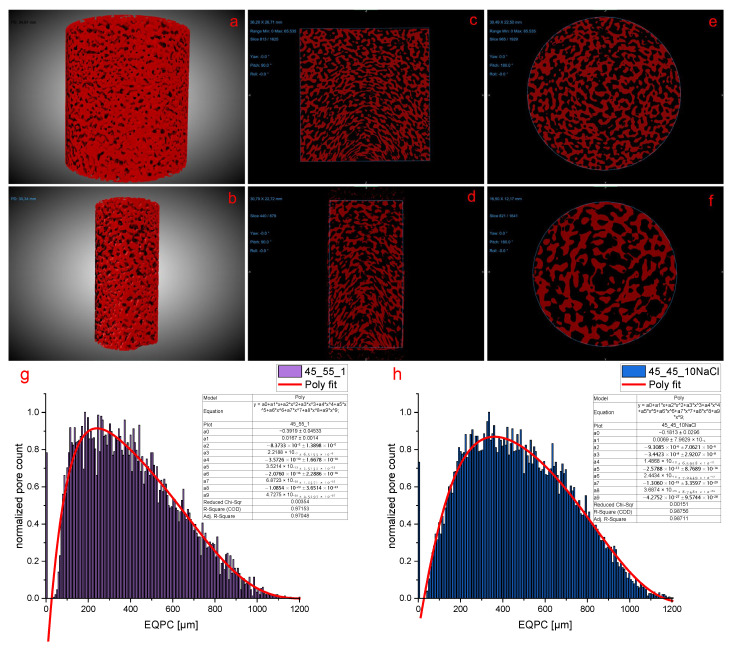
XRM images of 45_45_10NaCl and 45_55 (6 h annealed) scaffold cylinders with 3 cm height, and 2 cm and 1 cm diameter, respectively. (**a**,**b**) Three-dimensional models in x-y perspective, (**c**,**d**) vertical x-y plane from a middle slice, and (**e**,**f**) horizontal z plane from a middle slice. Normalized pore diameter distribution derived from equivalent circle (EQPC) of a 45_55 (**g**) and a 45_45_10NaCl (**h**) scaffold as a 256 binned plot (7.9 µm column width) with polynomial fitting (excluding pore sizes < 38 µm).

XRM was used to generate 3D models of a 45_55 and a 45_45_10NaCl scaffold to collect data on porosity (Figure 4). This allowed the characterization of the overall morphology across a bigger scaffold area and to compare the previously obtained results from SEM, HG porosimetry, and general composition of the blends.

The calculation of the void space of the whole 45_55 (Figure 4b) and 45_45_10NaCl scaffold yielded 54% and 53% void space, respectively. This is in line with the amount of PEO present in the initial blend and the uncertainty introduced by manual segmentation during evaluation. The x-y slices (Figure 4c,d) showed a deformation of the morphology, which is most pronounced at the lower edge of the image.

The XRM samples had to be annealed in open crucibles in order to maintain similar ventilation conditions as within the aluminum sandwich apertures to secure comparability. The deformation originated from the blend material creeping up the aluminum crucibles. However, this should not affect overall porosity, and in the upper part, which is next to the closed part of the crucible, there is no deformation at all (Figure 4c,d). 

As the samples in the sandwich aperture have direct contact with the aluminum plates across almost the entire blend material, it should be safe to say that very little deformation should be observed. This is in line with the observation made in SEM, where deformation is visible only in the outer 5 mm part of the scaffold disk.

XRM data revealed the pore size distribution beyond the Hg porosimeter’s 215 µm limit (Figure 4g,h). Both the 45_55 and 45_45_10NaCl scaffolds showed a tailing distribution with pore sizes up to 1200 µm, peaking at 225 and 350 µm, respectively. However, both scaffolds did not show any pores between 4 and 38 µm but a significant number of small pores below 4 µm, which were not or were only hardly accessible via Hg intrusion. Overall, the data obtained via XRM complement the data from Hg porosimetry.

Since the PCL is subjected to mechanical, thermal, and radiation stress in many steps during the production of the scaffold, it is necessary to track the molecular weight during the manufacturing process, as the molecular weight correlates with many physico-chemical and mechanical properties (Table 3). Overall, the molecular weight is weakly influenced by either the processing in the internal mixer and during annealing, or by electron beam sterilization (25 kGy). M_w_ showed a small increase in molecular weight across all processing steps, but regarding the small number of samples examined (*n* = 3) and the standard deviation being of the same magnitude as the increase, it is considered insignificant. The small decrease from granulate to scaffold of 7 kDa in M_n_, however, is significant (two-sample *t*-test, * ≤ 5%). Furthermore, a small increase in M_n_ after electron beam sterilization was observed (two-sample *t*-test, *p* = 0.049).

The crystallinity of the scaffold’s PCL could not only play an important role regarding the feasibility of co-crystallizing CS-g-PCL on its surface but is also well known to impact general properties of thermoplastics, including mechanical stability and degradation behavior.

**Figure 5:**
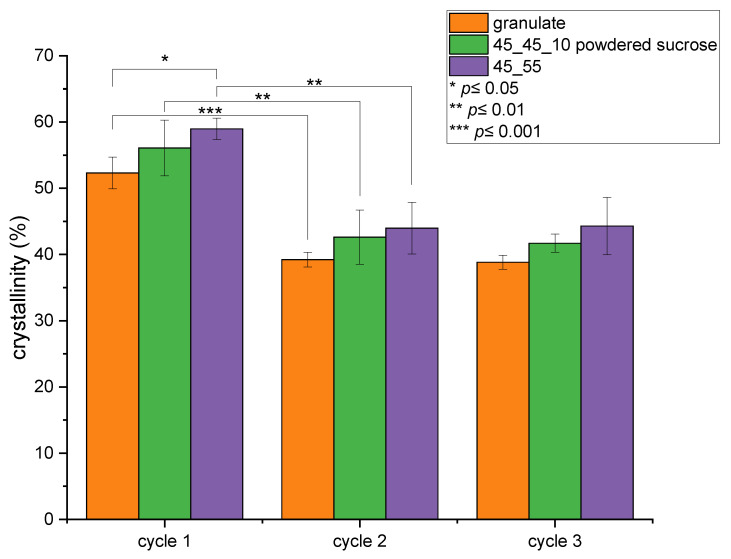
Crystallinity in % of raw granulate, 45_55 and 45_45_10P scaffolds obtained from DSC heating cycles.

The crystallinity of PCL granulate and two different scaffold species was derived from the measured enthalpy of fusion divided by the enthalpy of fusion of completely crystalline PCL (139.3 J/g [56]) with the former measured via DSC across three cycles (Figure 5). The crystallinity derived from the first melting cycle is most interesting as it originates from the thermal history introduced by the material processing steps. In order to evaluate the significance of apparent changes in crystallinity, a two-sample t-test was performed with a significance level of at least * ≤ 5%. All further results stated as significant satisfy this criterion. Bauer et al. have shown that the processing of PCL into textile fibers via melt spinning has no significant influence on the crystallinity [29]. The 3D porous scaffolds showed an increase in crystallinity when comparing granulate and 45_55 scaffold cycles, which was statistically significant. On the other hand, the increase for samples with powdered sucrose species was not statistically significant. In cycles 2 and 3, with the thermal history completely eliminated, the general trend of higher crystallinity in the porous structures compared to the granulate PCL was not statistically significant. Generally, the crystallinity was significantly lowered, which is in line with the findings on melt-spun textile PCL of Bauer et al. [29].

### 3.2. Synthesis of CS-g-PCL_x_

**Figure 6:**
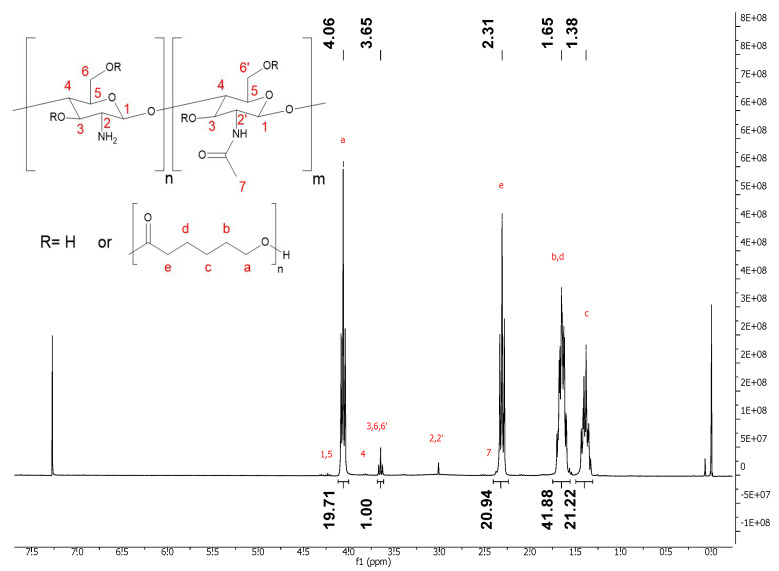
^1^H-NMR of CS-g-PCLx=_29_ for calculation of the ratio “x” via comparison of the respective integrals a and 3,6,6′ from PCL and chitosan, respectively. Measured at 600 MHz in CDCl_3_ with TMS at RT.

Synthesis of CS-g-PCLx was performed in a slightly modified procedure of de Cassan et al. [33]. Two different ratios “x” of PCL units per glucosamine unit were obtained by adjusting the amount of ε-caprolactone added (CS-g-PCL_29_ and a CS-g-PCL_56_). The ratio in the resulting graft polymer was calculated from ^1^H-NMR spectra comparing signal “a” representing two protons of a PCL’s carbon against the signal of three protons from glucosamine groups (positions 3 and 6, or 3 and 6′ for the acetylated unit, respectively) (Figure 6). Ratio “x” was calculated via:

x = ((1/2 · Aₐ)) / ((1/3 · A₃,₆,₆′))

The amount of PCL units per glucosamine unit was always determined to be lower than the amount calculated from the reaction mixture. Theoretical stoichiometric calculation led to ratios x = 24 and 72 with ε-caprolactone (M = 114,14 g/mol, D = 1,08 g/mL) and Chitosan (DDA 83%, M = 168,47 g/mol). This was also true for other ratios and batches synthesized. The difference between theoretical and analyzed ratios remained similar for a chitosan batch but had to be redetermined for each batch. All further studies presented here were performed with graft copolymers derived from the same chitosan batch.

### 3.3. Surface Modification via Self-Induced Crystallization

**Figure 7:**
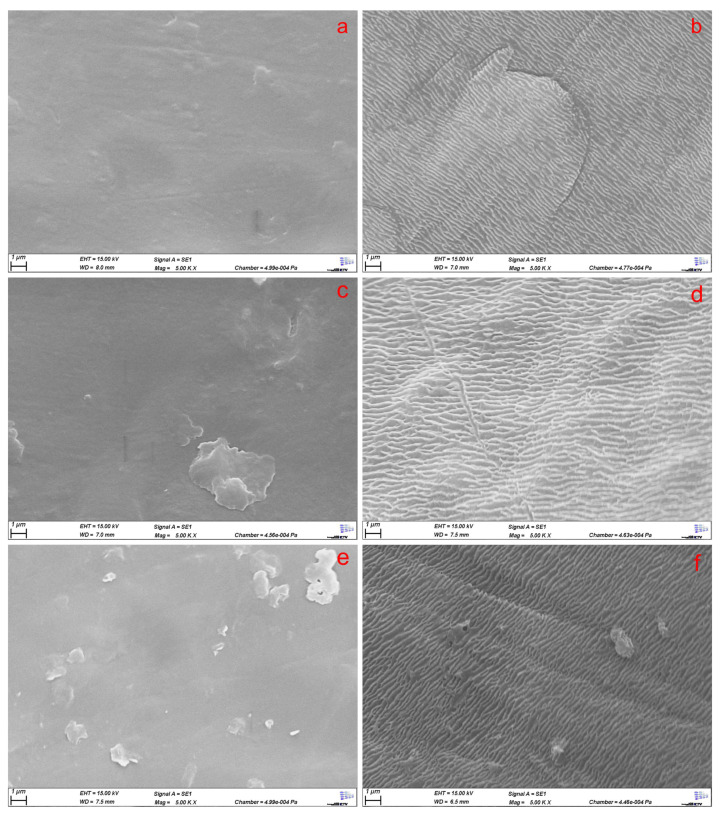
SEM images of 5000× magnified scaffolds with (**a**) 45_55, (**c**) 45_45_10NaCl, (**e**) 45_45_10P unmodified and (**b**,**d**,**f**) as their CS-g-PCL_56_ modified counterparts.

As already shown for electrospun PCL fibers and melt-spun textiles by de Cassan et al. [33] and Bauer et al. [29], respectively, CS-g-PCL can be used to coat PCL surfaces. The graft polymer is bound to the surface by a co-crystallization of the PCL side chains at the surface, resulting in the chitosan determining the surface properties of the coating [33]. The coating with CS-g-PCL resulted in a change in surface morphology. Investigating several different 3D porous PCL scaffolds via SEM showed that the dip coating with CS-g-PCL induced a similar change in surface morphology (Figure 7).

**Figure 8:**
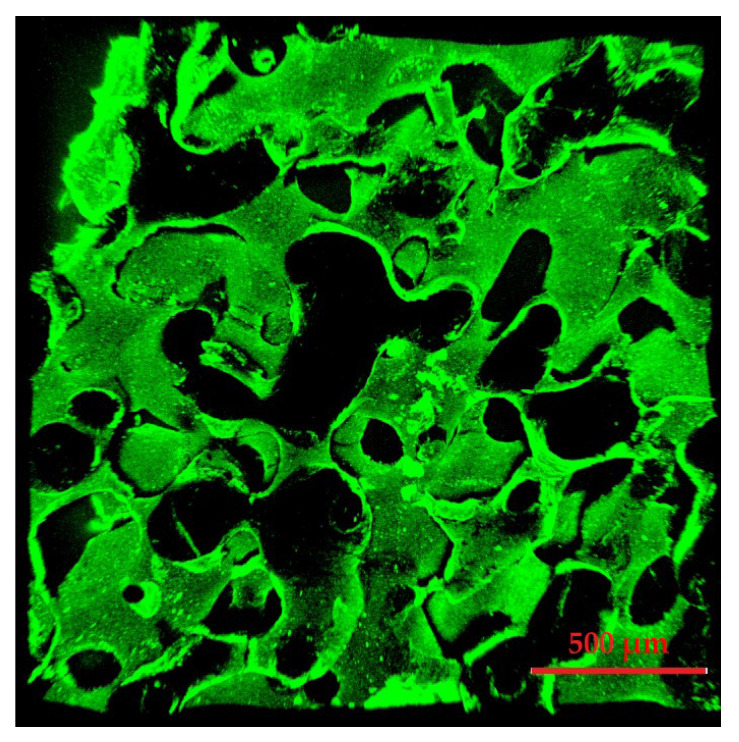
3D CLSM image of a 45_55 scaffold modified with CS-g-PCL_56_/alginate fluoresceinamine with 200 µm depth.

The grooves created were consistent across the whole scaffold, indicating a homogenous coating. However, a change in surface morphology could also solely originate from the etching of AcOH during the dip coating process. To clarify that the change was due to a CS-g-PCL coating and, indeed, that Chitosan determines the surface properties, a further analysis was conducted. Similar to de Cassan et al. [33], Bauer et al. [29], and Emonts et al. [17], a fluoresceinamine-modified alginate was attached to the surface of scaffolds modified with CS-g-PCL_56_ and investigated via CLSM (Figure 8).

**Figure 9:**
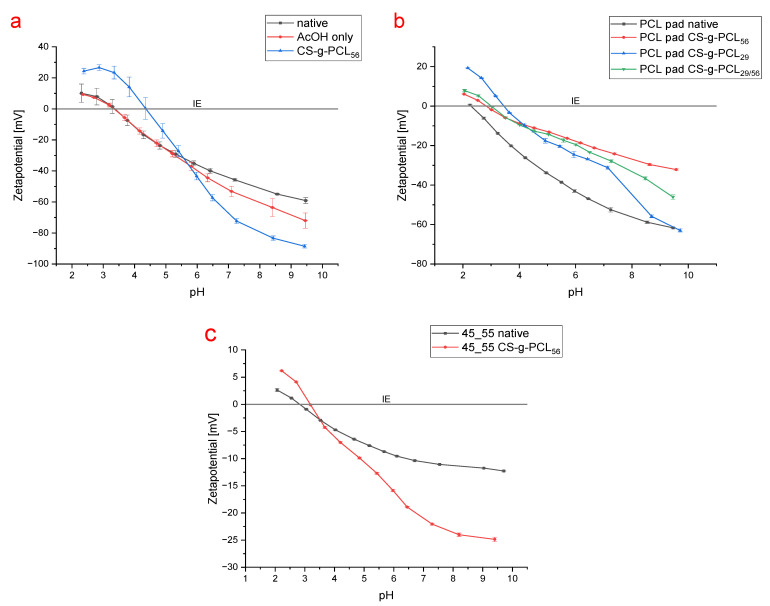
Zeta potential in dependence of pH of (**a**) native, AcOH only, and CS-g-PCL_56_-treated electrospun PCL fiber mats, (**b**) native and CS-g-PCL_x_-modified PCL pads (non-porous), and (**c**) native and CS-g-PCL_56_-modified 45_55 porous scaffolds.

Coverage was homogeneous across all samples. Non-CS-g-PCL-treated scaffolds showed no fluorescence, indicating that the modification is necessary for alginate application and, furthermore, opening the path for a CS-TPP nanoparticle-based release system. Further proof the surface morphology change due to modification can be found in the comparative surface potential analysis of non-, AcOH-, and CS-g-PCL-treated samples (Figure 9).

### 3.4. Surface Potential Analysis

As the analysis of CS-g-PCL-modified scaffolds via CLSM and SEM can only provide indirect insight into the feasibility and possibilities of the modification, it is far from being a quantitative analysis. In order to fill this gap, we analyzed different PCL samples regarding their surface potential (zetapotential) as a function of the pH. For comparison, in addition to the native porous PCL, AcOH- and CS-g-PCL-treated electrospun fiber mats were investigated across a range from pH = 10 to 2 in a gap cell (Figure 9a). The intention was to investigate whether and how much the CS-g-PCL modification impacts the surface potential, especially compared to AcOH-only treatment as it is the solvent used in the dip coating process. Native PCL fiber mats showed a negative zetapotential trend overall towards higher pH values, only reaching positive values below an isoelectric point (IEP) at pH = 3.3. A similar trend was observed in other studies under similar conditions on diverse porous PCL scaffolds [57,58]. The sole treatment with AcOH just decreased zetapotential slightly for pH values above 5.5. On the other hand, the CS-g-PCL-modified samples showed a different course of the surface potential as a function of the pH. It showed a s-shaped characteristic, with a significantly higher zetapotential below pH = 5.5 and a lower zetapotential above.

With solid PCL pads (non-porous) in a gap cell, the overall zetapotential increased significantly across the whole pH range when proceeding from native PCL to CS-g-PCL_56_-modified material (Figure 9b). With CS-g-PCL_29_-modified pads, the increase in zetapotential was even more pronounced at pH < 4 while being less intense above pH = 4. However, at a higher pH (>9), there was no difference observed in zetapotential compared to unmodified material.

When loading one side of the gap cell with CS-g-PCL_29_-modified pads and the other with a CS-g-PCL_56_-modified sample, the resulting zetapotential values were, as expected, in between those of pure CS-g-PCL_56_- and CS-g-PCL_29_ samples.

For the measurement with interconnected 3D porous PCL samples, a special measuring cell for porous materials was used; all other parameters were adopted from the gap cell measurements. After modification with CS-g-PCL_56_, the 45_55 species showed a clear change in surface potential across almost the whole pH range (Figure 9c). Above pH = 3.5, the zetapotential dropped to significantly lower values, whereas above pH = 3.5 the trend was inverted.

### 3.5. TGF-β_3_ Release Monitoring of via ELISA

**Figure 10:**
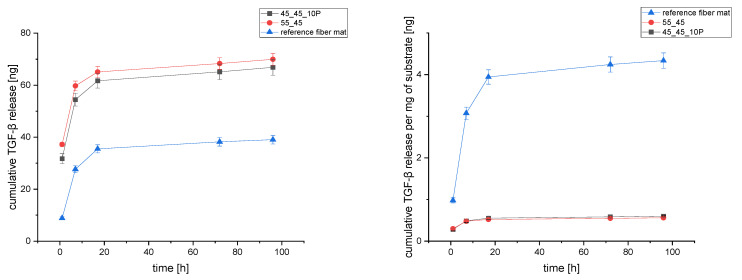
Cumulative TGF-β_3_ in vitro release in absolute (**left**) and per mg of scaffold (**right**) from 1 to 96 h.

For the investigation of a possible drug delivery system installed on the inner and outer surfaces of the porous PCL scaffolds, TGF-β_3_ was incorporated into chitosan/tripolyphosphate nanoparticles [49]. The porous PCL scaffolds modified with CS-g-PCL and subsequently with alginate were treated with the nanoparticle suspension. This procedure resulted in coatings loaded with TGF-β_3_. The release of TGF-β_3_ from CS-g-PCL/alginate/CS-TGF-β_3_-TPP-modified 3D porous PCL was monitored over 32 days (1, 8, 25, 96 h onwards until day 32 in 5-day intervals) via ELISA and depicted as cumulative release amount against time (Figure 10). As a reference, the well-investigated loading and release from electrospun PCL fiber mats by Sundermann et al. [49] and Berten-Schunk et al. [54] were treated and monitored accordingly. The release from both the 45_45_10P and 55_45 scaffolds outperformed the amount released from the reference fiber mats by a factor of 2. However, if the weight of the different substrates is taken into account, the reference fiber mats outperformed both scaffolds by a factor of 5.8. This is because of the very high surface-to-volume ratio of electrospun fiber mats. Nevertheless, this advantage due to the higher specific surface area is irrelevant for the desired application as an implant, with the electrospun fiber mats being limited in thickness due to technical limitations in manufacturing.

### 3.6. Cell Culture Vitality Assay

**Figure 11:**
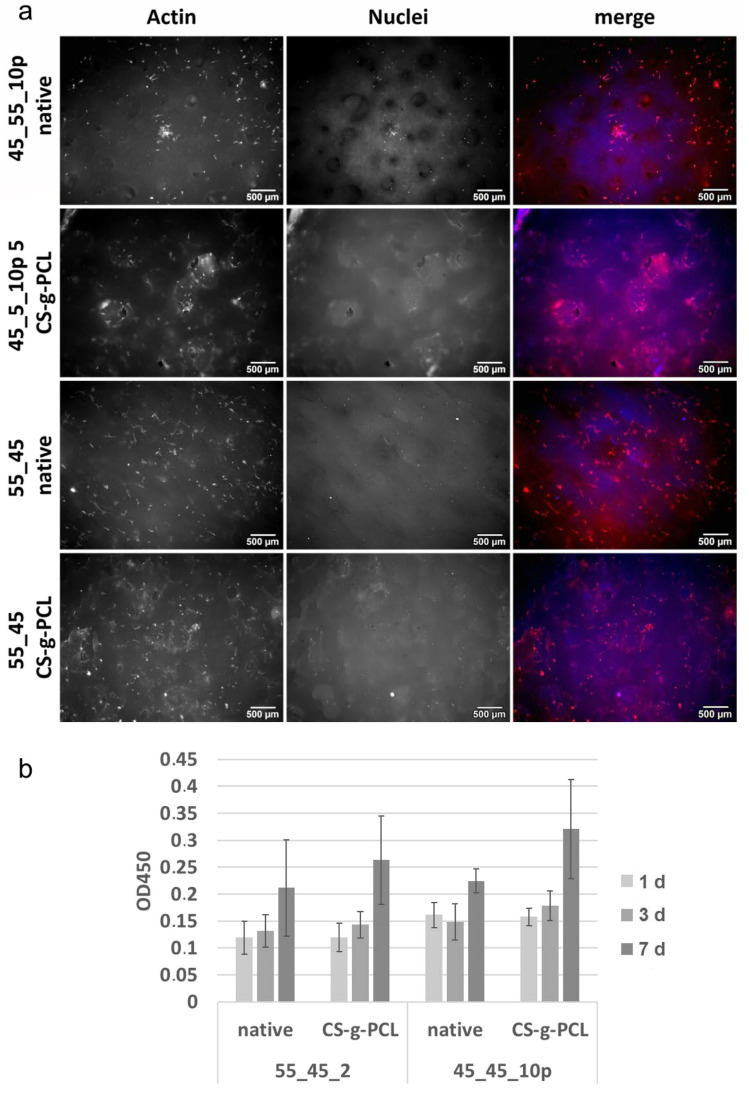
In vitro cell culture experiment. In (**a**) phalloidin (red) and DAPI (blue) staining is shown with separate nuclei and actin staining (white). Scale bar: 500 µm. In (**b**) the vitality of MSCs is shown over time (24 h, 3, and 7 days).

For cell culture assays, two different scaffolds were chosen (Figure 11). A standard 55_45 scaffold and a more delicate 45_45_10P scaffold in order to compare the performance based on their different inner morphologies. MSCs were seeded on both types of scaffold, and the vitality was determined after 1, 3, and 7 days. The morphology of the cells, which survived and proliferated on the scaffold surface, was analyzed after 3 days of incubation and was stained with phalloidin (red) for the actin cytoskeleton and DAPI (blue) for the DNA. Cells, which survived, proliferated on and migrated into these scaffolds. The CS-g-PCL coating did not seem to have a negative impact on the cells. This observation coincides with the vitality assay. Over the observation period of seven days, the cells proliferated, and an increase in vitality could be determined.

## 4. Discussion

There are numerous requirements for in situ tissue-engineered scaffolds across an interdisciplinary field of professions and tissues, each with completely different properties needed. Adjusting the pore size of a scaffold as well as the release of specific growth factors from it could improve vascularization and cell ingrowth [18,19,54]. Scaffolds can be obtained in various ways, such as electrospinning, melt spinning, or 3D printing. However, adjusting the pore size of scaffolds obtained via these techniques is difficult and often limited. For example, although electrospun fibers offer a high surface-to-volume ratio, their inter-fiber space is mainly in the sub-micrometer region, hindering cell ingrowth and vascularization [33]. Here, the interconnected 3D porous PCL scaffolds obtained via the combined blending, annealing, and leaching process present an attractive alternative. This process and various variations of it, like foaming and freeze-drying approaches, have already been proven to yield promising scaffold designs [24,26,27]. However, most studies have not gone beyond this and only adapted the material itself without the introduction of surface modifications or the release of active ingredients.

By combining for the first time the 3D porous PCL prepared via co-continuous polymer blends according to Mehr et al. [24] with a coating with CS-g-PCL graft polymer previously used by Jing et al. and de Cassan et al. [33,48] on nanoscale electrospun PCL-based fibers, porous scaffolds with tailored surface properties on the one hand, and a drug release system on the other hand, were obtained. This system was exemplarily investigated for TGF-β_3_ as a growth factor. The released amount of TGF-β_3_ was high and sustained enough to possibly induce cell differentiation [54].

The fabrication of the interconnected 3D porous PCL scaffolds with tailored pore size distribution yielded reproducible results, and the substitution of the PEO with NaCl, fine, and powered sucrose was successful. Pore size and distribution were tailored according to the required size of 100–250 µm for optimal cell ingrowth and vascularization [18,19]. With 4-6 h annealing, it even exceeded the required pore sizes. This suggests that a scaffold with tailored pore size could be produced even faster and more mechanically robustly due to a higher PCL content. The sucrose and sodium chloride-containing species showed increased porosity and a more delicate structure than their PCL/PEO-only counterparts. In addition to the macropores, the number of micropores observed in the pure sucrose scaffolds could be increased by an appropriate design, which would further increase the surface area, and thus possibly also the amount of active ingredient to be released. Adding CS-g-PCL_56_ directly into the blend for cutting the graft copolymer modification step from the process, as de Cassan et al. [35] did for electrospun fibers, did not work. The heat during mixing and especially annealing appears to cause the graft copolymer to act as a compatibilizer for PCL and PEO, reducing phase separation and thus eliminating the mechanism that leads to co-continuous phases [59]. On the other hand, the thermal and mechanical stress during blending of standard 45_55 blends did not significantly affect the molecular weight. Only a small decrease in M_n_ from granulate to scaffold was observed, which is expected as mechanical and thermal stress during mixing and annealing are known to decrease polymer chain lengths. After electron beam sterilization, the M_n_ rose slightly, which is in line with the findings of de Cassan et al. [55]. However, the increase is close to insignificant (two-sample, *t*-test, *p* = 0.049). This is important for the design and prediction of the degradation behavior, as it suggests that these necessary production steps do not negatively impact the materials properties.

The overall fabrication process took 7 min for blending, 6 h for annealing, and 5 days of leaching, with the latter having the potential to be reduced significantly with a higher, more directed solvent flow. Although it took more time for a full fabrication sequence compared to alternative approaches like electrospinning, the whole process was much better scalable and not as complex and error-prone as electrospinning [60]. 

Furthermore, the easy tuning of the scaffold size made the annealing process favorable in that regard, as electrospun scaffolds are limited in thickness due to inherent technical limitations.

Therefore, they typically yield thinner, less mechanically stable scaffolds than comparable scaffolds from approaches similar to this study [26,27].

As the CS-g-PCL modification is based on the co-crystallization on a PCL surface, the degree of crystallinity could play an important part and was therefore investigated via DSC. The overall degree of crystallinity of a 45_55 scaffold (55–58%) was close to the absolute values observed by de Cassan et al. [55] for electrospun PCL fiber mats. The crystallinity increased slightly during fabrication. However, the degree of crystallinity is not the only factor to consider, as there are others, like the size of surface crystallites, which could have an impact on the co-crystallization process [29,61]. The decrease observed with the 45_45_10P species, which contain powdered sucrose, could be due to finely encapsulated sucrose cavities within the PCL. Since these could not be reached during leaching, they contribute to the total mass, thus diluting the PCL and therefore apparently reducing the total crystallinity in DSC measurements.

The synthesis of two different CS-g-PCL_x_ (x = 29, 56) successfully extended the series of species described by de Cassan et al. [33] and Jing et al. [48], promising more chitosan dominated surface properties with the x = 29 species. These differences were shown in pH-dependent zeta potential measurements, and it was made clear that the modification is responsible for the change. The presence of the graft copolymer was indicated in SEM images across a variety of scaffolds from different blend compositions. The coating was further visualized using CLSM as homogenous films, as has been shown previously for coated electrospun fiber mats [33]. The possibility to install the fluorescent alginate implied a decent loading and release capability with TGF-β_3_ from a nanoparticulate release system. In fact, the release of TGF-β_3_ from scaffolds was possible and the amount released was higher than the control group of electrospun fiber mats already known from de Cassan et al. [33]. The absolute amount burst released over 96 h from 45_45_10P and 55_45 scaffolds was 67 ng and 70 ng, respectively, with both values being equal within the margin of error. According to Roger et al. [53], a concentration of 5–10 ng/mL in in vitro was determined to be sufficient to induce cell differentiation and growth. Similar numbers were found in investigations by Berten-Schunk et al. [54], Barry et al. [62], and Mueller et al. [63]. On the other hand, the amount released expressed per mg of substrate was lower compared to the fiber mats. However, this should not really matter as the electrospun fiber mats are limited in thickness on the process side and therefore cannot compete in terms of the absolute amounts released and the mechanical properties required for potential in vivo applications. In a study from Bauer et al., which was part of the same release study referenced against the same electrospun fiber mat standard, the melt-spun textiles delivered similar amounts of TGF-β_3_ released per mg of substrate as the scaffolds in this study [29]. The burst release, which was also observed with the melt-spun textiles in Bauer et al., could further be compensated using blocking layers [64].

## 5. Conclusions

Tissue engineering with scaffolds from interconnected 3D macroporous PCL in combination with a trauma-specific nanoparticulate release system could offer a potential solution to overcome the limitations of current surgical treatments of the musculoskeletal system that rely on solely physical approaches without incorporation of active agents released from an implant. The versatile nature of the scaffold pore size distribution and the multifunctional capabilities of the CS-TPP release system offer a broad variety of possible applications. However, there are a lot of requirements for such a potential in situ TE material to meet. Mechanical, biological, and morphological factors must be addressed.

Two requirements were met: a cell type-specific pore size distribution and porosity, as well as the attachment of a surface modification that provides cell compatibility and a platform for a release system able to provide active agents. The amount of TGF-β_3_ released should be sufficient to induce cell differentiation with a possible therapeutic effect when used in vivo.

The fabrication of scaffolds from PCL with adjustable interconnected 3D pores is relatively straightforward, easy to scale, and convenient using inexpensive, abundant ingredients like PCL, PEO, sucrose, and NaCl. The pore size distribution of the scaffolds can be tailored via various means on a macro- and micropore level. The graft copolymer surface modification worked well, and its homogeneous coverage was visualized via CLSM, and the impact on surface potential was shown in zeta potential measurements.

The CS-g-PCLx can also be tailored to change its properties depending on the average length x of PCL side chains attached to the chitosan backbone, thus influencing the surface potential of the implant material. The amount of graft copolymer needed for modification is comparably low, as only a small amount is needed for the dip coating process to be successful. The response of MSCs as observed by morphology, adhesion, and viability tests positively confirmed cytocompatibility towards both the native and graft copolymer-modified scaffolds, with no indication for the modification to impede cell attachment and growth.

## Figures and Tables

**Table 1:** Blend compositions in vol.-% and processing parameters.

No.	PCL	PEO	CS-g-PCL_56_	Fine Sucrose (F)	Powdered Sucrose (P)	NaCl	Temperature [°C]	Processability
1	45	55	/	/	/	/	100	Very good
2	50	50	/	/	/	/	100	Very good
3	55	45	/	/	/	/	100	Very good
4	40	55	5	/	/	/	100	Decent (granulose)
5	35	55	10	/	/	/	100	Decent (granulose)
6	25	55	20	/	/	/	100	Decent (granulose)
7	35	/	/	65	/	/	175	Very good
8	45	/	/	55	/	/	175	Very good
9	55	/	/	45	/	/	175	Very good
10	45	50	/	5	/	/	100	Very good
11	45	45	/	10	/	/	100	Very good
12	45	25	/	30	/	/	100	Not processible
13	45	/	/	/	55	/	175	Very good
14	45	45	/	/	10	/	100	Very good
15	45	50	/	/	/	5	100	Good (slightly granulose)
16	45	45	/	/	/	10	100	Good (slightly granulose)
17	35	45	/	/	/	20	100	Decent (granulose)
18	45	25	/	/	/	30	100	Not processible

**Table 2:** CS-g-PCL_x_ synthesis overview.

No.	CS [mg]	ε-Caprolactone [mL]	Yield [g]	Theoretical x in CS-g-PCL_x_	Calculated x in CS-g-PCL_x_ via ^1^H-NMR [48]
1	350	5.3	4.80	24	29
2	350	15.9	12.07	72	56

**Table 3:** Molecular weight measured via GPC (relative styrene standard) of PCL in different processing states.

Processing Step	Mn [kDa]	Mw [kDa]
granulate	104 ± 2	173 ± 4
45_55 scaffold	97 ± 2	175 ±4
45_55 scaffold sterile	103 ± 3	178 ± 2

## Data Availability

The original contributions presented in this study are included in the article and Supplementary Material. Further inquiries can be directed to the corresponding authors.

## Supplementary Materials

The following supporting information can be downloaded at: https://www.mdpi.com/article/10.3390/polym17030383/s1, Figure S1: ^1^H-NMR of a CS-g-PCL_56_ species.
