# Supplementary material for: 3D Porous Polycaprolactone with Chitosan-Graft-PCL Modified Surface for In Situ Tissue Engineering
Source: Polymers (Basel). 2025 Jan 30;17(3):383. doi: 10.3390/polym17030383 (PMC11820431; doi:10.3390/polym17030383)
Supplement: Supplementary file 1 [file polymers-17-00383-s001.zip › polymers-3383209-supplementary.pdf]

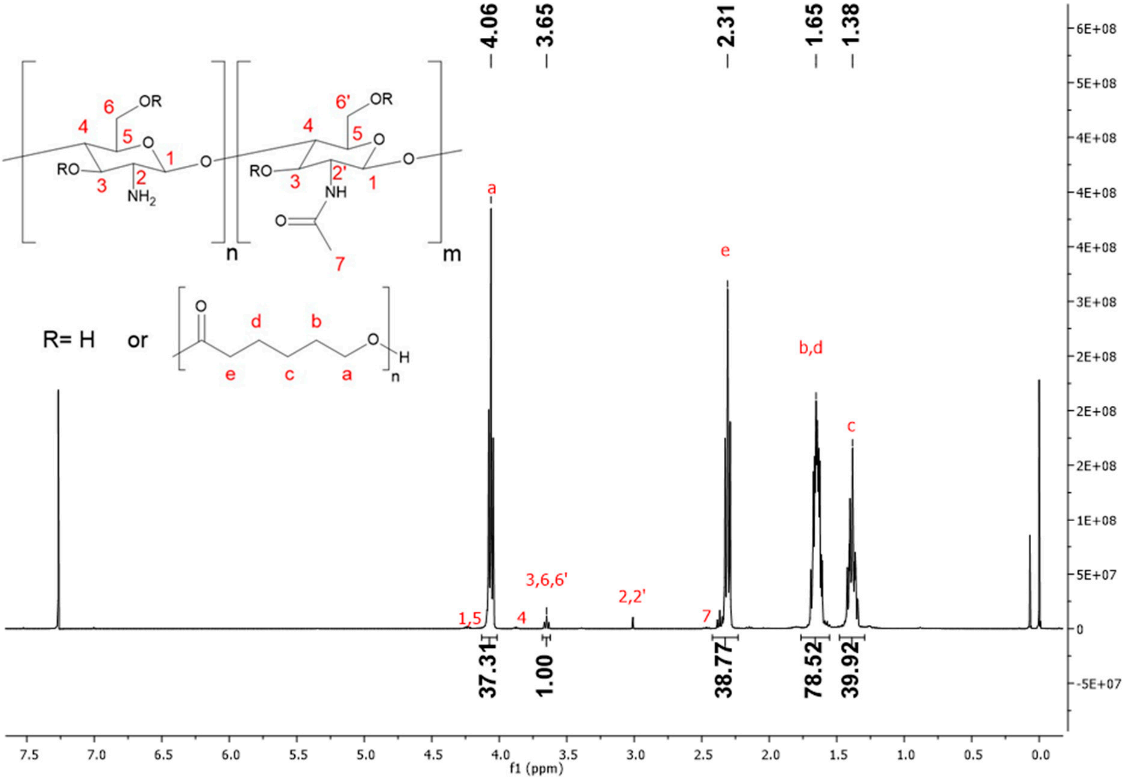

Figure S1. ^{1}H-NMR of CS-g-PCL$_{x=56}$ for calculation of the ratio “x” via comparison of the respective integrals a and 3,6,6′ from PCL and chitosan, respectively. Measured at 600 MHz in $CDCl_3$ with TMS at RT.
